# Experimental implementation of the peripheral nerve block clinical registry: an observational study

**DOI:** 10.3389/fmed.2025.1486300

**Published:** 2025-01-27

**Authors:** Ali Dabbagh, Firoozeh Madadi, Moein Ebrahimi, Shideh Dabir, Maryam Vosoughian, Mastaneh Dahi, Mohammadreza Moshari, Soudeh Tabashi, Mohsen Ariannik, Zahra Azizi

**Affiliations:** Anesthesiology Research Center, Shahid Beheshti University of Medical Sciences, Tehran, Iran

**Keywords:** peripheral nerve block, regional anesthesia, analgesia, safety, registries

## Abstract

**Background and aims:**

Peripheral nerve block (PNB) is commonly used, but there is a lack of data on its effectiveness and safety in the clinic. Therefore, anesthesiologists have limited insight into how they are faring in terms of both safety and efficacy. Additionally, No PNB registry is recorded in IRAN. Herein, we reveal how a hospital might use information gathered in a hospital registry of PNB outcomes to guide future quality enhancement efforts.

**Method:**

This was an observational, prospective, and unicenter study of all peripheral nerve blocks done in the operating room since December 22, 2022. After studying the data collected by the existing registries worldwide, the anesthesiology research center at Taleghani Hospital developed a questionnaire that incorporated the varying parameters set forth by earlier research and registries. Parameters were documented for each patient.

**Results:**

A total of 105 patients, were accrued from December 22, 2022, to July 23, 2023. The brachial plexus blocks namely axillary, infraclavicular, and popliteal blocks were the most frequently used PNBs, respectively. The indications that generated the greatest demand for PNBs were upper limb fractures, I&D, and amputation, respectively. 82.9% of blocks were conducted while patients were sedated employing systemic administration of sedatives. In this registry, there were no deaths or complications with sequelae. The median duration of hospitalization following admission to the hospital was 3 days. The mean patient satisfaction score was 9.46 out of 10.

**Conclusion:**

Our analysis demonstrates the effectiveness, safety, and feasibility of peripheral nerve blocks in preoperative anesthesia. It is recommended to continue the registry and conduct additional studies to enhance our understanding of this procedure.

## 1 Introduction

Peripheral nerve block (PNB), a form of regional anesthesia or analgesia, is useful for a variety of common procedures. It is applicable to surgeries in the upper and lower limbs, abdomen, and thorax. Studies have shown that peripheral nerve blocks are preferable to general anesthesia because they reduce side effects and improve clinical outcomes (1, 2). Some new studies have also provided novel techniques, such as sonography guidance (3) and systemic or local adjuvants, that enhance the quality of care provided by PNB. Such progressions have boosted PNB’s popularity (4).

PNB improves clinical and financial outcomes (5). PNBs are more effective at the management of postoperative pain, thereby reducing the need for analgesics after a variety of surgical procedures (6). Restricting opioid use reduces the likelihood of negative outcomes and has public health implications (7). PNBs also improve postoperative recovery, utilization of hospital resources, and patient satisfaction (4). The advantages of PNB, such as reduced hospitalization time and decreased occurrence of complications, are correlated to a lower financial burden on society, hospitals, and the healthcare system (8, 9). In the past few decades, PNBs have grown in popularity due to their beneficial features. Modern technological advances have enabled continuous perineural catheter-administered local anesthetic infusions and ultrasound-guided needle insertion (3).

The expanding use of PNB in healthcare facilities and outpatient clinics has uncovered additional problems and difficulties such as side effects and complications (10). In addition, despite its numerous benefits, PNB is still a novel concept in developing countries, particularly low-income and middle-income nations (11, 12). This study aimed to investigate the patient demographics and evaluate the outcomes of PNB in the context of a developing country.

## 2 Materials and methods

### 2.1 Designing the study

The foundation of this local registry began on December 22, 2022. This is the report of registry sharing of data and outcomes of PNB to evaluate health care quality. This registry comprises demographic and procedural information regarding patients. We conducted a prospective observational study at Taleghani Hospital. We continued the standard treatments for the patients. All the treatments and procedures were conducted in accordance with the latest guidelines and indications. Our team closely monitored and documented the progress of patients without making any alterations to their therapeutic plans. The design and report of this study align with previous guidelines for observational studies (13). The research was approved by the ethical committee of Shahid Beheshti University of Medical Sciences (Ethical code: IR.SBMU.RETECH.REC.1401.694). Our study is in accordance with the principles set forth in the Helsinki Declaration. We took informed consent from all patients. The patients who refused to participate in our study were excluded. Our study was in accordance with the Health Insurance Portability and Accountability Act of 1996 (HIPAA).

### 2.2 Study sample

From December 22, 2022, to July 23, 2023, all PNBs performed by anesthesiologists or anesthesiology residents at Taleghani Hospital were included. The anesthesiologist administering the block along with the nurse anesthetist in charge of the patient filled out a questionnaire and continued the patient’s follow-up until discharge from recovery. Each anesthesiologist’s information was compiled on paper and then entered into a database.

After surgical procedures, patients were contacted and evaluated for post-operative satisfaction and potential complications. This function is typically performed by a research assistant, general practitioner, or anesthesiology resident.

### 2.3 Data parameters

The date of surgery and PNB, age, weight, height, BMI, and gender were included among the demographic data. The past medical history, drug history, addiction, history of chronic pain, type of surgery, duration of surgery, duration of hospitalization, type of block, the extent of sensory and motor block, person who performed block, needle type and size, drugs utilized to block and drug concentration, volume, adjuvants, sedatives, time to perform the block, time to the beginning of the full block, time for the beginning of pain and satisfaction score of patient and surgeon were all collected. Our time scale for beginning to pain and satisfaction score was limited to discharge from the recovery room.

For each patient, the presence or absence of possible side effects was also recorded by an anesthesiologist or resident of anesthesiology (9 anesthesiologists and 24 residents of anesthesiology). Possible side effects included paresthesia, nausea, vomiting, cardiac arrest, ischemic heart disease, arrhythmia, headache, vertigo, blurred vision, auditory problems, hematoma, paralysis, or hemothorax. There was a space for additional side effects not listed here. Complications reported after discharge were not included. If a complication or side effect was detected during hospitalization, it was monitored until resolution. Puncture wound infection was not recorded as a complication.

Block success and whether it was converted to general anesthesia were also documented. A failed block was defined as one that was placed but did not result in any noticeable analgesia or blockade.

### 2.4 Statistical analysis

To ensure accurate analysis, we adhered to the recommendations outlined in previous guidelines for analyzing clinical research (14). A software environment for statistical computation, SPSS version 27 was utilized to conduct statistical analyses. The purpose of this investigation was to quantify the frequency of cases that received at least one type of block. In order to enhance the readers’ understanding of the work, we employed statistical metrics such as mean, median, and interquartile range (IQR) in our study.

## 3 Results

### 3.1 Patients

Hundred and five regional blocks were performed during the initial seven-month study period from December 22, 2022, to July 23, 2023. The demographic information is briefed in Tables 1, 2. There were no mortality or major complications.

**Table 1:** Summary of registry.

Variable	Category	Number	Percent
Gender	Female	33	31.4
	Male	72	68.6
Type of addiction	No addiction	88	83.8
	Alcohol	1	1.0
	Amphetamine	1	1.0
	Cannabis	2	1.9
	Methadone	5	4.8
	Morphine + cocaine + cannabis	1	1.0
	Opium	6	5.7
	Oxycodone	1	1.0
Chronic pain	No	100	95.2
	Yes	4	3.8
	Yes (during hospitalization)	1	1.0
Motor block	Complete	78	74.3
	No motor block	15	14.3
	Partial	12	11.4
Who did block	Professor	14	13.3
	Professor + resident	3	2.9
	Resident	88	83.8
PGY of residents	1	9	8.6
	2	35	33.3
	3	29	27.6
	4	15	14.3
Drug of block	Bupivacaine	1	1.0
	Lidocaine	88	83.8
	Lidocaine + bupivacaine	16	15.2
Result of block	Partially successful	1	1.0
	Successful	101	96.2
	Unsuccessful	3	2.9
Side effects	No	96	91.4
	Yes	9	8.6
List of side effects	Paresthesia	2	1.9
	Nausea	3	2.9
	Headache	3	2.9
	Dizziness	2	1.9
	Ptosis	1	1.0

Descriptive analysis. Part 1. (PGY, The grade of a resident; MIN, minute).

**Table 2:** Summary of registry.

Variable		Minimum	Maximum	Mean	Median	Percentiles			IQR
						25	50	75	
Age		13.00	91.00	47.02	47	31	47	64	33.5
Height (cm)		138.00	195.00	169.24	170	161	170	176.25	15.25
Weight (kg)		55.00	140.00	75.55	75	65	75	80	15
Duration of surgery (min)		15.00	360.00	100.76	90	60	90	120	60
Duration of Hospitalization (day)		1.00	27.00	5.34	3	2	3	7	5
Volume (cc)		10	50	35.98	40	30	40	40	10
Sedation	Midazolam (mg)	1	5	1.51	1	1	1	2	1
	Fentanyl (μg)	50	300	84.00	100	50	100	100	50
	Dexmedetomidine	50	1,000	176.92	100	100	100	150	50
	Propofol (mg)	20	50	30.00	25	20	25	45	25
	Ketamine	4	20	12.00	12	4	12	-	-
Duration of doing the block (min)		2	20	8.77	10	5	10	10	5
Time of onset of the block (min)		0	60	7.32	5	5	5	8	3
Time of onset of the full block (min)		0	25	11.92	10	10	10	15	5
Satisfaction score of patient (from 10)		0	10	9.46	10	10	10	10	0

Descriptive analysis. Part 2. (CM, centimeter; KG, kilogram; MIN, minute; mg, miligram; μg, microgram).

### 3.2 Types of blocks

In these cases, the nerve blocks were classified into three main groups: 1. Brachial plexus blocks including axillary, costoclavicular, infraclavicular, supraclavicular, and interscalene approaches for block, 2. Lower limb blocks including popliteal, femoral, and ankle blocks, and 3. Truncal blocks [i.e., transversus abdominis plane (TAP) block]. Types of blocks are summarized in Table 3.

**Table 3:** Types of blocks in the registry.

Location of surgery	Types of blocks	Number	Total Number	Percentage
Abdomen	TAP block	3	3	2.9
Lower limb	Ankle	2	23	21.9
	Femoral	1		
	Femoral + Popiteal	8		
	Popliteal	10		
	TAP	1		
Upper limb	Axillary	52	79	75.2
	Axillary + infraclavicular	2		
	Costoclavicular	1		
	Infraclavicular	17		
	Supraclavicular	8		
	Supraclavicular + Interscalen	1		

TAP, transversus abdominis plane.

Seventy nine blocks were performed in the upper limb, 23 blocks in the lower limb, and three in the abdominal area. Blocks in the limbs were done for anesthesia and blocks in the trunk were done for analgesia. Three of the four trunk blocks were performed for abdominal surgeries and one was performed for hip surgery. The axillary block was performed on 52 patients (49.5%) and was the most frequent block. Following that, infraclavicular and popliteal blocks were the most prevalent blocks. The costoclavicular block which is not a part of our residency curriculum had the lowest frequency (<1%). All blocks performed in our center were done by ultrasound guidance except the ankle block which was performed using anatomic landmarks. Ankle blocks were landmark-guided.

With a nerve block frequency of 55.2%, the fracture was the surgical indication with the highest proportion of cases receiving a nerve block. It was then followed by I&D, which had an 8.6% block frequency. Indications of PNBs are summarized in Table 4.

**Table 4:** Indication of block.

Location of surgery	Department	Surgery	Number	Total number	Percentage
Trunk	Obsteristic surgery	Myomectomy	1	3	2.9
		Transabdominal hysterectomy	2		
Lower limb	Orthopedic surgery	Total hip arthroplasty	1	18	17.1
		I&D	6		
		PCP	2		
		Fracture	2		
		Amputation	5		
		Foreign body	1		
		Abscess	1		
	Vascular surgery	I&D	3	5	4.8
		Amputation	1		
		Foreign body	1		
Upper limb	Orthopedic surgery	Fracture	40	71	67.6
		PCP	15		
		CTS	1		
		Finger graft	1		
		Finger repair	1		
		Tendon repair	1		
		Plaque placement/removal	4		
		Amputation	2		
		Laceration	5		
		Mallet finger	1		
	Vascular surgery	AVF	4	7	6.7
		Aneurysm in arm	1		
		Anastomosis of hand vessels	1		
		Amputation	1		
	General surgery	I&D	1	1	1

AVF, Arteriovenous fistula; CTS, Carpal tunnel release surgery; I&D, incision and drainage; PCP, percutaneous pinning.

### 3.3 Blocks without motor block

Seventy-eight PNBs (74.3%) resulted in motor block. Twelve blocks (11.4%) resulted in a partial motor block. In 15 PNBs (14.3%), no evidence of motor block was found. Five popliteal blocks, 4 ankle blocks, 4 femoral + popliteal blocks, and 1 costoclavicular block comprise partial blocks. Four popliteal blocks, 4 femoral + popliteal blocks, 1 femoral block, 4 TAP blocks, and 2 axillary blocks comprise PNBs without motor block.

### 3.4 Drugs used in this study

Lidocaine and Bupivacaine were used for PNB. Three main local anesthetic therapeutic regimens, including lidocaine (83.8%), bupivacaine (1%), and a combination of lidocaine and bupivacaine (15.2%), were recorded.

Epinephrine was the most commonly utilized adjuvant. In 59% of cases, only epinephrine was utilized. In 2.9% of cases, Dexamethasone was given as a single adjuvant. Epinephrine and dexamethasone were combined in 2.9% of cases.

### 3.5 Success rate and satisfaction

A successful PNB was described as a block that resulted in a complete sensory block in the region innervated by the blocked nerve. Partially successful PNB is characterized by a blockage of a nerve that results in an incomplete loss of sensation in the region innervated by that nerve. An unsuccessful PNB is characterized as a block that fails to result in any sensory block in the region innervated by the blocked nerve. Hundred and one PNBs (96.2%) were successful. One PNB (1%) was partially successful and 3 PNBs (2.9%) were totally unsuccessful. Among these, the mean satisfaction score was 9.46 (range: 0–10).

### 3.6 Side effects

**Figure 1:**
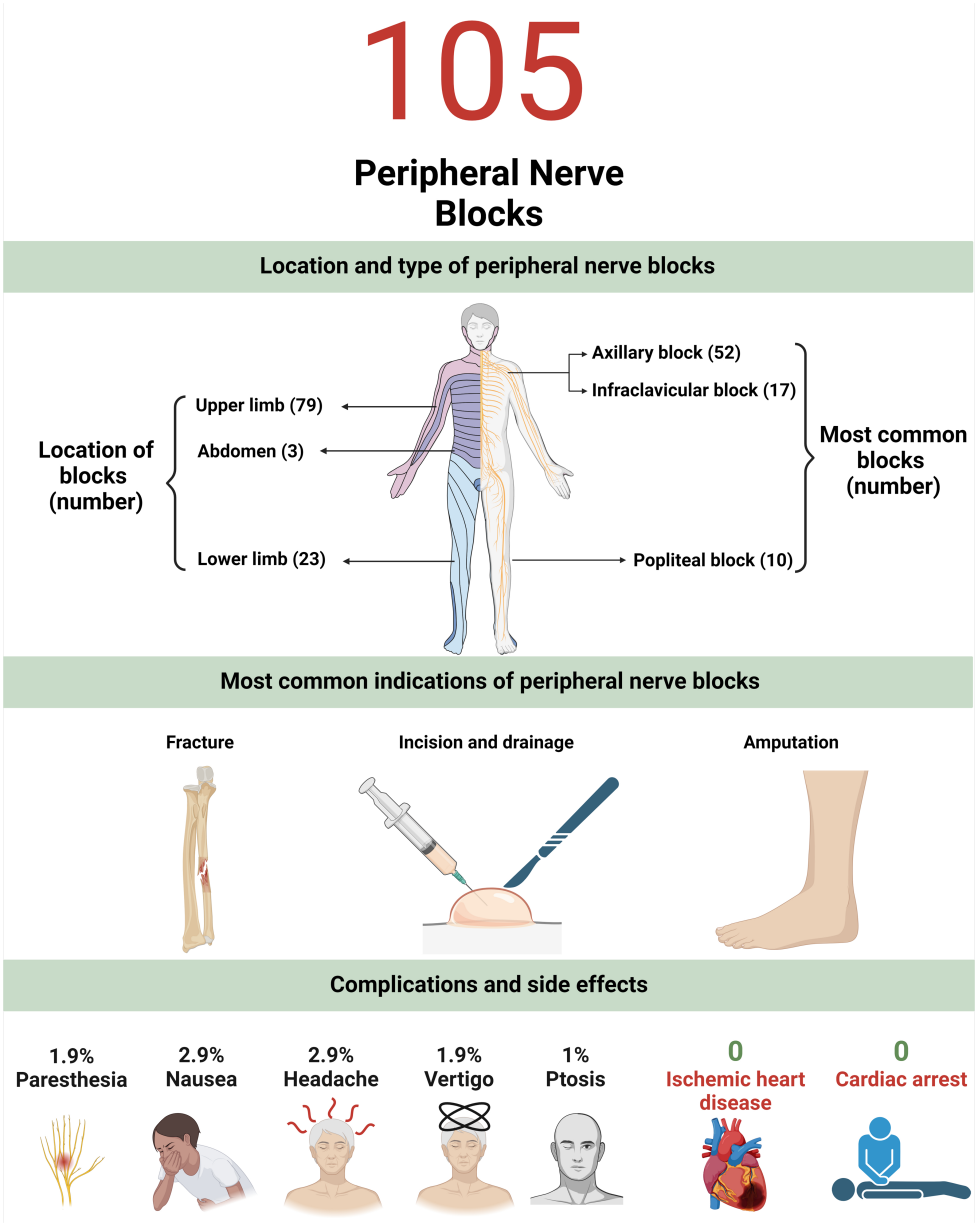
This figure summarizes the location and type of peripheral nerve blocks, most common indications and associated complications.

Nine patients (8.6%) experienced adverse events. In this study, patients experienced paresthesia, nausea, headache, vertigo, and ptosis at rates of 1.9, 2.9, 2.9, 1.9, and 1%, respectively. No patient experienced vomiting, cardiac arrest, ischemic heart disease, arrhythmia, blurred vision, auditory problems, hematoma, paralysis, or hemothorax (Figure 1).

## 4 Discussion

In this prospective study, we utilized information from a local database to report the frequency with which various PNB techniques were used during 105 surgical procedures. Axillary, infraclavicular, and popliteal PNB were the most prevalent types. Our findings are useful for better defining healthcare practice patterns, trends, and disparities. This analysis revealed that PNB is safe, with an extremely low incidence of complications. Our research also indicates that PNBs are frequently used and widely accepted for various types of surgical procedures, particularly orthopedic procedures. Since the foundation of this registry, our questionnaire’s layout has undergone several minor modifications which were made to enhance questionnaire clarity and usability.

PNBs are gaining popularity worldwide. To our knowledge, to date, there is no registry about PNB in Iran. This study was conducted as the first stage toward establishing a national registry of PNB in Iran. Regional anesthesia provides significant prospective improvements in perioperative and postoperative outcomes, such as superior postoperative analgesia, reduced risk for postoperative complications, improved recovery phase, and decreased hospitalization time.

The majority of PNBs were performed by residents. PNBs performed by residents included a variety of PNB types to improve the materials of their training. These PNBs include ankle, axillary, costoclavicular, femoral, infraclavicular, popliteal, supraclavicular, interscalene, and TAP blocks. Residents in our registry perform a greater number of PNBs than residents in other centers (15). This was done based on prior evidence demonstrating that the use of an ultrasound guide decreases the time required for the placement of a block and reduces complications when the block is performed by a resident (16). Given our good safety record and limited occurrence of complications at our center, the greater number of PNBs performed by residents indicates that we can enhance their educational experience through the use of ultrasonography and professor supervision. Implementing this approach can enhance the proficiency and effectiveness of residents in performing PNBs, perhaps minimizing errors and complications when they practice as independent physicians after completing their education.

In our research popliteal and combined popliteal and femoral motor block account for the majority of PNBs with partial motor block or no motor block at all. The absence of a motor block may result from multiple factors. The first reason is the optimization of the drug concentration block. The higher the concentration of the blocking drug, the greater the motor block (17). Second, certain pharmaceuticals are superior to others in terms of motor block (18). These parameters should be considered because the neuroanatomy of the lower and upper limbs differs in terms of nerve conduction velocity (19). Perhaps an increase in the volume and concentration of block drugs, as well as the development of newer block drugs, could help us accomplish better motor nerve block (18).

The majority of PNBs conducted at our center used ultrasound independent of who did the block, the resident, or the professor. We did not use sonography as a guide for the ankle block. Because it did not help us perform the task more effectively. Numerous studies indicate that ultrasound is associated with better outcomes and a reduced incidence of complications, so its utilization is encouraged (16).

In this registry, lidocaine alone was the most commonly used drug for PNB. Local adjuvants were included in 64.8% of PNBs. The most prevalent adjuvant was epinephrine, which was added to the PNB syringe as an epinephrine wash. Dexamethasone was the second most commonly utilized local adjuvant in this study which is a widely available adjuvant that prolongs the duration of block (20).

In our study, the most prevalent sedative combination was midazolam and fentanyl. Recently, dexmedetomidine has been introduced as a safe sedative that enhances clinical outcomes in PNB (21). Dexmedetomidine was administered for sedation in 12.5% of PNBs in our registry.

The average length of hospitalization in our study was 5.34 days. This is longer than the length of stay reported in previous investigations; which may be explained by longer duration of hospitalization for patients who were admitted for vascular surgery. According to a study by Lenart et al. revealed that the average duration of hospital stays for patients with PNB following major orthopedic surgery is 3 days. The duration of stay for non-PNB patients was 8 days (22). Even though the duration of stay in our study was 5.34 days, which is shorter than the length of stay of patients who did not receive PNB in other studies (22). Perhaps because of the longer duration of preoperative evaluation in Iran. However, additional research is required to optimize this technique in our country. This is one of the significant factors identified in our registry that highlights the deficiency of the optimal clinical application of PNB. If the number of individuals registered increases, it is possible that additional factors, such as sedation regimen, the combination of local medications used in PNB, and PNB-specific expertise, would be analyzed.

The average duration to do the block in our registry was 8.77 min. This is significantly higher than the data reported by high-income nations such as the United States. In the United States, the average time to perform PNB under ultrasound guidance was 1.8 min (16). This disparity may be due to the performers which were mostly residents.

The mean patient satisfaction score in our registry was 9.46, which is consistent with previous studies (23) that reported high patient satisfaction. This result indicates patient satisfaction in our clinical practice. This evidence demonstrates that it is possible to achieve high patient satisfaction ratings in low-income and middle-income countries by attempting to use PNB as a safe technique in surgical procedures.

Safety was another priority. 8.6% of patients had minor adverse effects. These side effects include paresthesia, dizziness, nausea, headache, and ptosis. No major complications such as nerve injury, catheter infection, hemorrhage, hematoma, or cardiac arrest have happened in our registry. This suggests that PNB is safe in our education and practice system. This safety enables the extension of the use of PNBs in ambulatory surgical procedures and for patients at high risk for general anesthesia. In this regard, Polshin et al. found that PNB reduces the length of hospital stays following ambulatory operations. This association was most pronounced during prolonged surgical procedures. PNB also decreases the need for opioids (24), which have unique adverse effects including apnea (25). This shows that PNB can replace general anesthesia in some cases. This is particularly essential for high-risk patients undergoing general anesthesia. In this regard, research initially focused on the distal portions of the lower limbs, but the current trend is to investigate the proximal portions. In hand and wrist procedures, Hadzic et al. compared infraclavicular brachial nerve block to general anesthesia. Infraclavicular PNB was related to fewer adverse effects, better analgesia, and greater patient acceptance (26). It has also been expanded to include additional proximal procedures. The knee is one of the most studied body regions that has been subjected to this comparison. In a study by Hadzic et al., patients undergoing knee arthroplasty under PNB had a superior recovery profile than those receiving general anesthesia. The efficacy of PNB in comparison with general anesthesia has also been demonstrated in populations aged 65 and older (27). Recent studies have also demonstrated the effectiveness above the knee (28). This evidence highlights the potential for achieving acceptable safety standards in low-income and middle-income countries, encouraging the use of PNB as a safe method in surgical procedures. Despite the emergence of various challenges and concerns, including potential side effects and complications (10), our paper demonstrates that PNB remains a safe and effective approach. This safety can provide motivation for us to further implement this method in our country and may encourage other countries facing similar economic challenges to adopt this approach and achieve improved levels of healthcare, thereby contributing to the advancement of global health equity. This is significant because PNB represents a new concept in developing countries. Having positive experiences in various countries can provide motivation to further implement this approach.

### 4.1 Limitations

This short-term study was undertaken at one center. This project was an early concept for a national registry that would be sustained and expanded to include more centers. Due to staff limitations, we could not evaluate the patient until full-block recovery. Acute pain service visits outside the operating room will solve this problem.

This observational study was prospective. Due to the small study population, side effects may have been underestimated. Thus, we did not attempt to link these data to side effects. By means of this registry, notable adverse events that warrant additional research can be identified, and prospective, randomized trials can be designed in the areas that have been identified as areas of concern.

It is hard to determine if PNB causes reported side effects. For this aim, we must compare the occurrence of these side effects between a PNB-treated and non-treated group. Our work is observational, thus we are unable to attribute symptoms like nausea to PNB. Achieving this goal requires comparative investigations.

## 5 Conclusion

This study illustrates that PNB is an anesthetic technique with significant potential for widespread application in the clinical practices of developing countries. Future studies involving larger sample sizes and alternative methodologies, such as randomized controlled trial, are necessary for a comprehensive comparison with other methods of anesthesia. This observational study aimed to characterize the current situation and facilitate the initiation of future, larger studies. Some of the grounds for continuing this procedure are its safety, efficacy, and high patient satisfaction. Furthermore, the results of this study emphasize the significance of continuing of PNB registry to uncover more hidden aspects.

## Data Availability

The raw data supporting the conclusions of this article will be made available by the authors, without undue reservation.
